# An Elastase Inhibitor ShSPI from Centipede Attenuates Bleomycin-Induced Pulmonary Fibrosis

**DOI:** 10.3390/toxins17050213

**Published:** 2025-04-24

**Authors:** Xi Lian, Bin Liu, Dan Li, Xinyao Wang, Chengbo Long, Xing Feng, Qiong Liao, Mingqiang Rong

**Affiliations:** 1 College of Life Sciences, Hunan Normal University, Changsha 410004, China; lianxi202230154001@hunnu.edu.cn (X.L.); liu.bin@hunnu.edu.cn (B.L.); lidan20040904@163.com (D.L.); wangxinyao@hunnu.edu.cn (X.W.); rongmq@hunnu.edu.cn (M.R.); 2 Chengdu PDBio Co., Ltd., Chengdu 610225, China; longchb@163.com; 3 Key Laboratory of Study and Discovery of Small Targeted Molecules of School of Pharmaceutical Sciences, Hunan Normal University, Changsha 410013, China; 4 Hunan Provincial Key Laboratory of Animal Intestinal Function and Regulation, Hunan Normal University, Changsha 410004, China; 5 The National & Local Joint Engineering Laboratory of Animal Peptide Drug Development, College of Life Sciences, Hunan Normal University, Changsha 410004, China

**Keywords:** idiopathic pulmonary fibrosis (IPF), elastase, centipede, neutrophils, inflammatory factors, ShSPI

## Abstract

Idiopathic pulmonary fibrosis (IPF) is a chronic lung disease characterized by the fibrotic thickening of the alveolar walls, resulting in compromised gas exchange, restricted ventilation, and respiratory failure. It has been indicated that elastase inhibitors reduced the severity of IPF by neutralizing excessive elastase levels in the lungs. ShSPI is an elastase inhibitor derived from centipede toxin. The present study evaluates the therapeutic effects of ShSPI in a bleomycin-induced idiopathic pulmonary fibrosis model. According to the results, ShSPI markedly reduced the weight loss, showing the improvement of health status in bleomycin-induced mice. Its robust antifibrotic effects were evidenced by the mitigation of alveolar structural damage, reduction in inflammatory cell infiltration, inhibition of collagen deposition, and suppression of fibrotic nodule formation. ShSPI effectively attenuated inflammatory responses by downregulating pro-inflammatory factors (IL-6, IL-1β, and MCP-1) and upregulating the anti-inflammatory factor interleukin-10 (IL-10). After delivered via inhalation, ShSPI exhibited favorable pharmacokinetic properties. It could be detected at 8 h at doses of 1 mg/kg and achieved maximum plasma concentrations (Cmax) of 188.00 ± 64.40 ng/mL in vivo. At high doses (160 mg/kg), ShSPI maintained a strong safety profile, with no detectable toxicity observed. This feature shows the therapeutic potential of ShSPI in the treatment of idiopathic pulmonary fibrosis and provides valuable evidence for its development as a novel peptide-based therapy.

## 1. Introduction

Idiopathic pulmonary fibrosis (IPF) is a chronic and progressive lung disease characterized by the thickening of the fibrotic alveolar walls, which leads to impaired gas transport, restricted ventilation patterns, and respiratory failure [1,2]. When the damage is severe, the repair process causes fibroblast proliferation, extensive collagen deposition, and irreversible fibrotic changes in the lung parenchyma [3,4,5]. The disease significantly impacts patients’ quality of life and reduces their life expectancy, with a median survival of only three years in the absence of treatment [6,7]. The late 2019 outbreak of novel coronavirus pneumonia (COVID-19) affected more than one hundred million people globally. The SARS-CoV-2 virus causes lung inflammation by infecting the lung’s target cells, thereby triggering an inflammatory cascade. This results in lung injury and secondary pulmonary fibrosis, which has been identified as the primary cause of death in this outbreak [8].

The pathogenesis of IPF is complex and can be broadly divided into three stages: injury–inflammation–repair. Dysregulation at any of these stages can contribute to the development of pulmonary fibrosis. In the initial stage, activated mesenchymal stromal cells and infiltrating inflammatory cells (neutrophils and macrophages, etc.) within the lungs secrete destructive proteases and a series of inflammatory factors, including tumor necrosis factor-α (TNF-α), macrophage inhibitory protein-2 (MIP-2), and C-X-C motif ligand 1 (CXCL-1). In the second stage, a significant number of fibroblasts are activated to produce extracellular matrix (ECM) and deposit it in the lungs. This process activates pathways like mitogen-activated protein kinase P38 (MAPK-P38), nuclear factor κB (NF-κB), Vascular Endothelial Growth Factor (VEGF), interleukin-6 (IL-6), interleukin-8 (IL-8), interleukin-13 (IL-13), and other inflammatory factors that are abundantly secreted. The third stage is characterized by a constant reiteration of the preceding two states, which ultimately leads to an overexpression of collagen, an excess deposition of ECM, a substantial production of matrix protein degradation products, and the formation of lung fibrosis. The formation of transforming growth factor-β1 (TGF-β1) and the Smad protein pathway activated by it, as well as the formation of α-smooth muscle actin (α-SMA), which is specific to myofibroblasts, serve as crucial indicators of pulmonary fibrosis formation [9,10].

Neutrophil elastase (NE) is a serine protease primarily released by neutrophils upon stimulation. It is the most released protease during injury because of the large number of neutrophils activated during injury [11]. Excessive NE activity has been observed in sputum or Bronchoalveolar Lavage Fluid (BALF) samples from patients with lung diseases, and it has been shown to be directly correlated with the severity of IPF [11,12]. NE can activate Toll-like receptor 4 (TLR-4) and the TLR4-mediated mitogen-activated protein kinase (MAPK) signaling pathway and further activate NF-κB through the Myeloid Differentiation Factor 88 (MyD88)-dependent TLR-4 signaling pathway, promoting the release of inflammatory factors such as TNF-α and IL-6, leading to an inflammatory cascade that is involved in the pathogenesis of lung diseases [13,14]. Therefore, the inhibition of NE activity may provide a novel therapeutic way for the treatment of IPF.

Bleomycins, used in our study to induce IPF, are a family of compounds produced by Streptomyces verticillis. It is able to cause cell damage independent from its effect on DNA by induction lipid peroxidation. This may be particularly important in the lung and in part account for its ability to cause alveolar cell damage and subsequent pulmonary inflammation. The lung injury seen following bleomycin comprises an interstitial oedema with an influx of inflammatory and immune cells. This may lead to the development of pulmonary fibrosis, characterized by enhanced production and deposition of collagen and other matrix components. Several polypeptide mediators capable of stimulating fibroblast replication or excessive collagen deposition have been implicated in this, but the precise role of these in bleomycin-induced fibrosis is yet to be demonstrated [15].

Currently, the two main drugs for IPF treatment, pirfenidone and nintedanib, can only delay the progression of the disease and do not provide a complete cure [16]. In addition, these drugs have undesirable side effects including nausea, vomiting, and severe diarrhea [17,18]. In contrast, peptide elastase inhibitors of natural origin act in a mostly reversible binding mode with NE and have a higher safety profile. The elastase inhibitor ShSPI, which is the subject of this study, is derived from the natural peptide centipede toxin. The mature peptide of ShSPI consists of 34 amino acids, with the sequence CPQVCPAIYQPVFDEFGRMYSNSCEMQRARCLRG. The formation of two pairs of disulfide bonds is essential for maintaining its structural stability. Compared with the typical Kazal structural domains, ShSPI lacks two cysteines and the corresponding disulfide bonds and belongs to the atypical Kazal family. With the help of an online tool, ShSPI has been predicted to contain a cystine-stabilized α-helix (CSH) motif formed by residues Ser-23 to Arg-33 and two antiparallel β-folded chains (Pro-11 to Asp-14, Gly-17 to Tyr-20). This structure gives ShSPI a stable spatial conformation, which facilitates its binding to and inhibition of elastase [19]. These properties give ShSPI a unique advantage in the development of elastase inhibitors. It brings new hope to patients with pulmonary fibrosis.

## 2. Results

### 2.1. Body Weight Changes in IPF Mice

**Figure 1:**
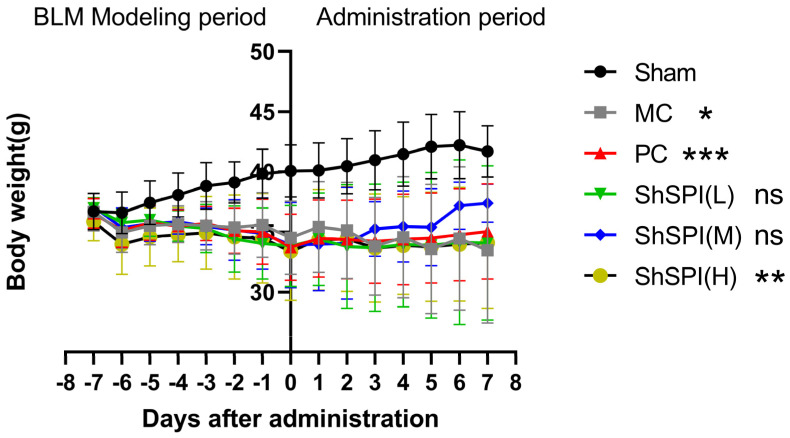
Plot of body weight changes in BLM-induced idiopathic pulmonary fibrosis (IPF) mouse models. The fold lines in the figure represent the body weight changes of mice in the sham-operated group (●), the model control group (■), the positive control group (▲), the ShSPI low-dose group (1 mg/kg, ▼), the ShSPI medium-dose group (2 mg/kg, ◆), and the ShSPI high-dose group (4 mg/kg, ○), respectively. * *p* < 0.05, ** *p* < 0.01, *** *p* < 0.001.

The body weight of each mouse was regularly examined, and the raw data were processed to derive the body weight changes in the BLM-induced idiopathic pulmonary fibrosis (IPF) mouse model (Figure 1).

Following the modeling intervention, a progressive reduction in murine body mass was observed, demonstrating a mean decrease of 13.61% during the 7-day experimental period (from modeling initiation to day 7 post-intervention). Notably, all experimental cohorts maintained physiological excretory functions and preserved normal feeding patterns throughout the study duration, with no observable abnormalities in metabolic homeostasis.

Throughout the drug administration period, the mice in the model group presented persistent emaciation and a curled posture. In contrast, the mice in the middle-dose ShSPI group (2 mg/kg) showed increased activity, while those in the high-dose groups (4 mg/kg) exhibiting a more favorable condition than those in the positive control group. These observations suggest that ShSPI has significant therapeutic efficacy.

Statistical analysis revealed that after modeling, the weight of the mice in the model control group (MC) continued to decrease until the 14th day, showing a significant difference compared to the sham-operated group (*p* < 0.001). After drug administration, the weight of the mice in the positive control group (PC) continued to increase until day 7, also showing significantly different from that of the sham-operated group (*p* < 0.001). After administration, the body weights of all mice in the ShSPI administration group continued to increase until day 7, demonstrating a significant treatment effect.

At the initial stage of modeling, the body weights of all the mice remained the same. Subsequently, the body weights of mice in the model control group decreased continuously from the 3rd day after modeling. After ShSPI administration, the body weight of mice in the positive control group and all ShSPI dose groups gradually rebounded. Specifically, the body weight of mice in the medium- and high-dose groups of ShSPI recovered more effectively, being comparable to that of the positive control group. The body weight of mice in the low-dose ShSPI group also showed an upward trend, albeit with a slower recovery rate.

### 2.2. ShSPI on Bleomycin-Induced Histopathological Changes in Lungs of Mouse Models

In this study, H&E staining was employed for structural observation, and Masson trichrome staining was utilized to detect collagen deposition. 

**Figure 2:**
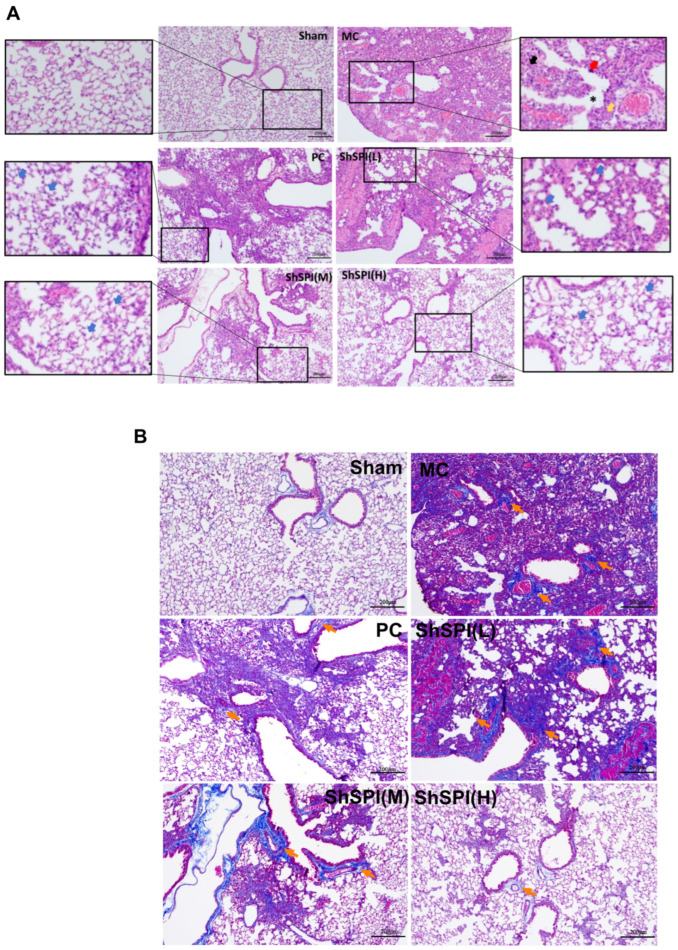
Histopathological changes in the lungs of IPF mice. (**A**) H&E staining reflects the histological structure and inflammatory infiltration of the lungs of IPF mice in each group. The lungs of model control (IPF) mice showed severe pathological changes, including thickening of alveolar walls (black↑), widening of lung septa (yellow↑), destruction of alveolar structure (*), and a large amount of inflammatory cell infiltration (red↑). After treatment with ShSPI, the degree of inflammatory infiltration in the lungs of mice was significantly reduced, and the alveolar structure was improved (blue↑). (**B**) Masson staining reflects the deposition of collagen fibers in the lungs of mice in each group. A large number of collagen fibers were deposited in the lungs of mice in the model control group (IPF), forming obvious fibrotic areas (orange↑). After treatment with ShSPI, collagen fiber deposition in the lungs of mice was significantly reduced, and the fibrotic area shrank.

Histological analysis using hematoxylin and eosin (H&E) staining revealed distinct morphological characteristics: Normal tissue specimens exhibited a well-preserved alveolar architecture with clearly demarcated septal boundaries, accompanied by negligible inflammatory infiltration. In contrast, in the model group, H&E staining revealed marked inflammatory infiltration and alveolar structure destruction. The bronchial epithelium exhibited signs of damage and detachment, accompanied by wall edema and arterial wall smooth muscle hyperplasia. Small foci of necrosis were also observed (Figure 2A). Regarding Masson staining, in normal lungs, collagen deposition was minimal. However, in the model group, Masson staining revealed a substantial collagen deposition in the pulmonary arterioles, septa, and terminal bronchioles (Figure 2B). The alveolar lumina were seen to be characterized by sheets of fibrotic nodules (fusion), and fused fibrotic nodules were observed within the alveolar structures (Figure 2B).

Treatment with ShSPI led to a substantial reduction in lung inflammation, a significant enhancement in alveolar structure, and a substantial inhibition of collagen deposition in the lungs of IPF mice. The optimal doses of the peptide drug ShSPI, administered via tracheal spray, for ameliorating idiopathic pulmonary fibrotic lesions appear to be 2 mg/kg and 4 mg/kg, while a dose of 1 mg/kg was less effective in addressing the model’s damage.

In conclusion, as demonstrated in the graphs, compared with the positive drug Pirfenidone, a certain dose of ShSPI also inhibited the lung injury, lung inflammatory infiltration, and fibrotic lesions in BLM-induced IPF mice. At the same time, its inhibitory effect was dose-dependent in the appropriate range, and 2 mg/kg was the lowest effective dose of the ShSPI peptide drug for the improvement of idiopathic pulmonary fibrotic lesions.

**Figure 3:**
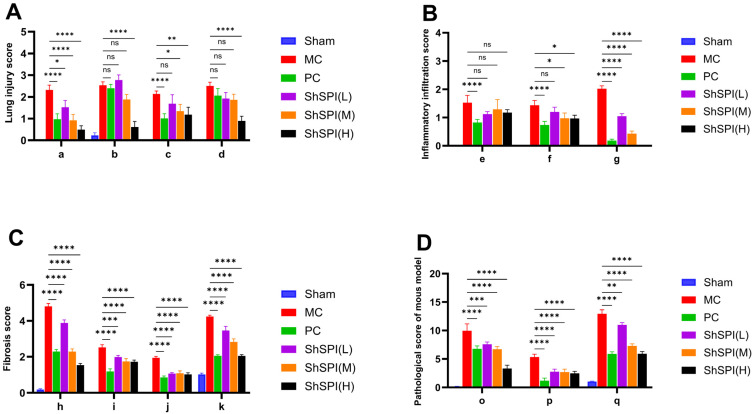
Pathologic scores of IPF mice: (**A**) lung injury score: a for edema, b for hemorrhage, c for terminal fine bronchial wall injury, and d for vascular injury; (**B**) inflammatory infiltration score: e for alveolar inflammatory cell infiltration, f for fine bronchiolar inflammatory cell infiltration, and g for macrophage aggregation; (**C**) fibrosis score: h for lung structural lesions, i for increased alveolar septa, j for alveolar epithelioid cell proliferation, and k for collagen deposition; (**D**) pathological score of mouse model: o is the pooled lung injury score, p is the pooled inflammatory infiltrate score, and q is the pooled fibrosis score. Data are expressed as mean ± SD. * *p* < 0.05, ** *p* < 0.01, *** *p* < 0.001, **** *p* < 0.0001. ns: not significant.

The severity of lung injury was quantitatively scored based on the extent of these changes above-mentioned (Figure 3).

### 2.3. Effect of ShSPI on Inflammation in Bleomycin-Induced Mouse Models

**Figure 4:**
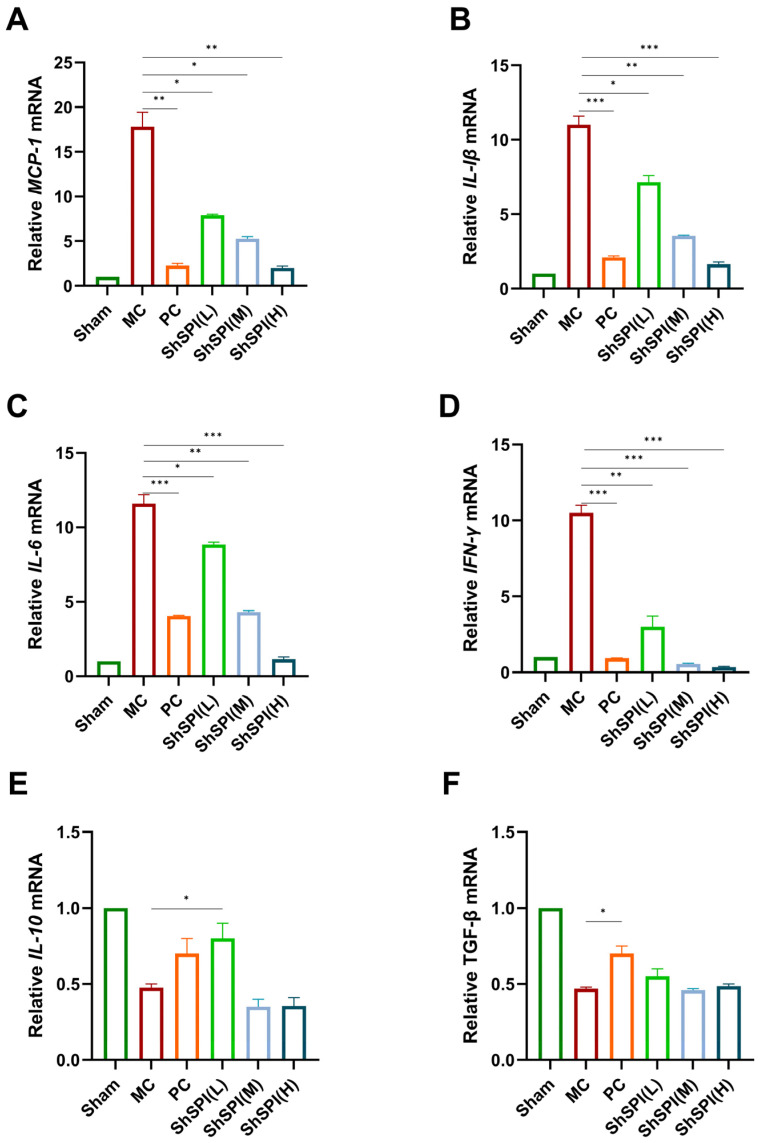
Release of inflammatory factors in lung tissue of IPF mice: (**A**) relative mRNA expression level of MCP-1; (**B**) relative mRNA expression level of IL-1β; (**C**) relative mRNA expression level of IL-6; (**D**) relative mRNA expression level of IFN-γ; (**E**) relative mRNA expression level of IL-10; (**F**) relative mRNA expression level of TGF-β. This figure demonstrates the effect of ShSPI on the mRNA expression of pro-inflammatory cytokines (MCP-1, IL-6, IL-1β, IFN-γ) and anti-inflammatory cytokine (IL-10) in lung tissues of bleomycin-induced IPF mice. ShSPI was found to significantly reduce the mRNA content of pro-inflammatory cytokines and restore the normal level of anti-inflammatory cytokine IL-10 by real-time fluorescence quantitative PCR. * *p* < 0.05, ** *p* < 0.01, *** *p* < 0.001.

“Cytokine storm” is a typical feature of idiopathic pulmonary fibrosis (IPF), with IL-1β being a key pro-inflammatory cytokine in the development. The present study investigated the potential of ShSPI to mitigate the release of inflammatory factors induced by bleomycin. RT-qPCR results demonstrated that bleomycin stimulation significantly increased the mRNA content of pro-inflammatory cytokines, including MCP-1, IL-6, IL-1β, and IFN-γ. The pirfenidone and ShSPI groups exhibited a reduction in these mRNA contents. Furthermore, ShSPI exhibited a concentration-dependent effect; with the increase in ShSPI concentration from 1 mg/kg to 4 mg/kg, the reduction in the mRNA levels of pro-inflammatory cytokines became more prominent. A high dose (4 mg/kg) of ShSPI restored these pro-inflammatory cytokine ShSPI mRNA levels to normal (Figure 4A–D). A low dose (1 mg/kg) of ShSPI restored the anti-inflammatory cytokine IL-10 mRNA levels to normal (Figure 4E). However, ShSPI had no significant impact on TGF-β mRNA content in mouse lung tissue (see below).

The results demonstrated that ShSPI significantly reduced the mRNA levels of these pro-inflammatory cytokines in a concentration-dependent manner. It is noteworthy that pirfenidone inhibits inflammatory cells (e.g., dendritic cells, macrophages, etc.) through multiple pathways, thereby reducing the release of a range of cytokines that mediate the inflammatory response [20]. When compared with the known anti-inflammatory drug pirfenidone, ShSPI also showed the ability to regulate the inflammatory response in vivo. Given that the concentration dosage of ShSPI (1–4 mg/kg) was significantly lower than that of pirfenidone (200 mg/kg) and that its ability to regulate the inflammatory response in vivo was similar, this suggests that the treatment of ShSPI is more efficient and safer. In summary, the findings indicate that ShSPI exerts substantial anti-inflammatory effects in the bleomycin-induced mouse IPF model, as evidenced by its capacity to markedly diminish the expression of pro-inflammatory cytokines and restore the normal level of anti-inflammatory cytokines.

### 2.4. Toxicity Study of ShSPI Single Administration on Mice

In the hematological indexes, the proportion of monocytes (Mon%) was higher than that in the blank control group and other dose groups at the dose level of 80 mg/kg. However, the abnormal changes of the aforementioned indexes did not show dose correlation, and other related indexes, such as the number of monocytes, did not show any abnormality. Therefore, the toxicological significance was not considered. The remaining indicators fell within the established reference ranges, and no statistically significant differences were observed among the groups (Table 1).

The results of blood biochemistry and electrolyte tests showed that the electrolyte indexes (ionized calcium and total calcium) were slightly lower than those of the blank control group at the dose levels of 40 and 80 mg/kg and that the electrolyte indexes (total calcium) were slightly lower than those of the blank control group at the dose level of 160 mg/kg. However, the magnitude of abnormal changes of the above indexes was very small, and they were within the range of the reference values; therefore, the toxicological significance was not considered. The rest of the indices were within the reference value range, and no significant difference was found (Table 2).

### 2.5. Pharmacokinetic Study of ShSPI in Mice

**Figure 5:**
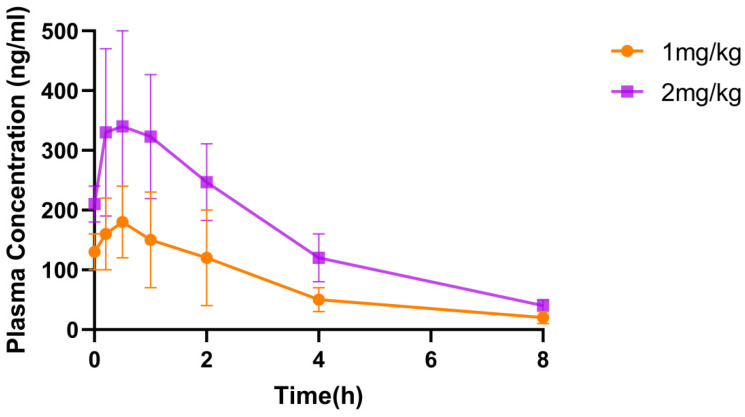
Blood concentration–time curve after drug administration in IPF mice. This figure demonstrates the changes in blood drug concentration over time in IPF mice after different doses of ShSPI were given by inhalation. ShSPI was rapidly absorbed in mice, and the blood concentration was higher and lasted longer in the high-dose group.

Each plasma sample was analyzed by LC-MS/MS, and the raw data were processed by DAS pharmacokinetic v2.1.1 software to obtain pharmacokinetic parameters (Table 3) and blood concentration–time curves (Figure 5).

In terms of in vivo exposure, ShSPI was detectable in vivo at 8 h post-administration with doses of 1 mg/kg and 2 mg/kg, achieving maximum plasma concentrations (Cmax) of 188.00 ± 64.40 ng/mL and 347.00 ± 151.00 ng/mL, respectively. However, by 24 h post-administration, the drug was no longer detectable. It is noteworthy that the data following tail vein injection of 1 mg/kg ShSPI were extremely challenging to analyze. The number of data points obtained was severely limited, with only three measurements available, which made any robust statistical analysis unfeasible. As a result, specific data for this injection route are lacking. This paucity of data indicates that ShSPI may be rapidly metabolized following tail vein injection, suggesting that this particular route of administration may not be suitable for ShSPI.

However, when considering the overall metabolism of ShSPI in mice through other assessment methods, it was determined that its metabolism is moderate. This balanced metabolic rate ensures that the drug remains in the body for a sufficient duration to elicit a therapeutic effect. Additionally, it effectively prevents toxicity enhancement associated with drug accumulation, likely due to its timely clearance from the body before significant accumulation can occur. This characteristic renders ShSPI a promising candidate for further development as a drug, provided that alternative and more suitable routes of administration are explored.

## 3. Discussion and Conclusions

Idiopathic pulmonary fibrosis (IPF) remains a challenging chronic lung disease with a limited number of effective clinical treatments. Pirfenidone and nintedanib, the currently approved antifibrotic drugs for idiopathic pulmonary fibrosis (IPF), have been shown to be effective in slowing lung function decline. Despite their therapeutic efficacy, the long-term clinical implementation of these medications is often associated with adverse effects that limit their chronic use [21]. For instance, diarrhea has been reported as the most common adverse event in patients treated with nintedanib [18]. Similarly, gastrointestinal and skin-related adverse events were more prevalent in patients receiving pirfenidone [16]. ShSPI, a novel elastase inhibitor, was investigated in this study with the aim of overcoming the adverse effect of existing drugs and providing a more effective and tolerable treatment for IPF.

The selection of the bleomycin-induced mouse model for pulmonary fibrosis was widely used. This model recapitulates multiple key aspects of IPF and other fibrotic interstitial lung diseases (ILDs), including patchy parenchymal inflammation, epithelial cell injury with reactive hyperplasia, epithelial–mesenchymal transition, fibroblast activation and differentiation into myofibroblasts, and damage to the basement membrane and alveolar epithelium. Its reproducibility and ease of induction make it an ideal choice for studying IPF [22,23]. Moreover, the model’s ability to mimic epithelial–mesenchymal transition and fibroblast activation is particularly relevant for studying the potential antifibrotic mechanisms of ShSPI, as these processes are key targets in IPF pathogenesis. During the course of the experiments, we conducted group allocation and drug administration on model mice to simulate clinical treatment scenarios. ShSPI effectively reversed the weight loss trend in IPF model mice, reflecting its potential to improve overall health and inhibit disease progression. Histopathological analysis of lung tissue showed that the peptide significantly alleviated pathological changes such as inflammatory infiltration, alveolar structure destruction, and collagen deposition, providing direct evidence of its antifibrotic effects. Furthermore, analysis of inflammatory factors indicated that ShSPI modulated the expression of inflammatory cytokines, reducing pulmonary inflammation and demonstrating dual mechanisms of anti-inflammatory and antifibrotic action.

Previous studies have showed that when nintedanib is administered orally, only a small NTD amount reaches the epithelial airway surface [24], necessitating high and frequent dosing and leading to serious systemic adverse effects [25]. In contrast, ShSPI exhibited a moderate metabolic rate in mice. These pharmacokinetic properties suggest that inhalation administration may be more suitable, as it can potentially achieve higher local concentrations in the lungs with reduced systemic exposure. It allows for more efficient delivery to the target site while minimizing the risk of systemic toxicity. Toxicity studies were conducted in mice by administering ShSPI at escalating doses up to 160 mg/kg. In contrast to nintedanib causing elevated liver enzyme levels in patients, ShSPI did not show significant toxic effects [26]. Histopathological examination of major organs revealed no pathological changes, providing a solid safety basis for further clinical translation. This peptide has some advantages compared to existing small-molecule elastase inhibitors. These properties suggest a wide range of potential applications in the treatment of IPF [27].

In conclusion, ShSPI has demonstrated a promising therapeutic potential and excellent safety in the treatment of pulmonary fibrosis, providing a new strategy for IPF therapy.

## 4. Materials and Methods

### 4.1. Peptide Synthesis and Refolding

ShSPI was chemically synthesized by GL Biochem (Shanghai) Ltd. (China) and subsequently refolded using standard protein purification techniques.

Peptides were refolded in a solution (pH 7.2) containing 10 mM glutathione (V900456, Sigma, Saint Loui, MO, USA) and 100 mM oxidized glutathione (V900363, Sigma, Saint Loui, MO, USA). The refolding solution was placed at 28 °C for 24 h. Then, we separated the different fractions using Reverse Phase-High Performance Liquid Chromatography (RP-HPLC) techniques and C_18_ column (Waters, Milford, CT, USA, 5 µm particle size, 250 mm × 4.6 mm). Matrix-assisted laser desorption/ionization time-of-flight mass spectrometry (MALDI-TOF MS, Autoflex speed TOF/TOF, Bruker Daltonik GmbH, Bruker Corporation, Saarbrücken, Germany) was performed to determine the purity of the peptides, and refolded ShSPI with purity higher than 95% was collected for further research [19].

### 4.2. Grouping and Administration of Model Mice

**Animal Model Preparation**: The peptide powder was dissolved in pre-cooled saline and filtered to remove bacteria, then a spray needle was attached to a micro-syringe to aspirate the peptide solution. A total of 56 ICR mice were anesthetized by intramuscular injection of avertin. After anesthesia, the mice were fixed supine, the tongue was gently pulled, the vocal folds were exposed with a laryngoscope, the trachea was gently inserted, and the nebulized solution was rapidly pushed and injected [28]. Each mouse was administered 100 μL of a 0.4 mg/mL bleomycin solution via tracheal spray, with the treatment performed once. Seven days after modeling, the mice were randomly grouped for ShSPI treatment based on weight loss scores. An additional 8 mice received the vehicle (normal saline) and were designated as the sham group [29].

**Grouping**: Seven days after modeling, mice that exhibited a 10–15% reduction in body weight were selected. A total of 48 suitable mice were randomly divided into five groups: the model control group (MC), the positive control group (PC), and the ShSPI low-dose groups (1 mg/kg), medium-dose groups (2 mg/kg), and high-dose groups (4 mg/kg).

**Drug Administration**: Starting on day 7 post-modeling, ShSPI peptide treatment groups were anesthetized with intramuscular avertin and received tracheal spray administration of the peptide. The low-dose group was treated with 1 mg/kg ShSPI peptide, the medium-dose group with 2 mg/kg, and the high-dose group with 4 mg/kg. The positive control group, without anesthesia, received 200 mg/kg pirfenidone (IPFD) via oral gavage. All groups were treated once daily for seven consecutive days.

**Body Weight Monitoring**: The body weight of the mice was monitored and recorded from the modeling phase to the end of treatment (a total of two weeks).

**Sample Collection:** Twenty-four hours after the final treatment, all ICR mice were euthanized via intramuscular injection of suforane. The left upper lobe of the lung from each mouse was collected and immediately immersed in 4% paraformaldehyde fixative for 48 h. The fixed tissues were then processed for H&E and Masson staining.

### 4.3. Histopathologic Analysis of the Lungs

The samples from experiment 4.1 were then processed using standard protocols for paraffin embedding, and 5 μm thick paraffin sections were prepared. Sections were stained with hematoxylin–eosin (HE) and Masson’s trichrome stains [30,31]. Pathological changes in lung tissue were observed under a light microscope. Multiple fields were randomly selected, and the severity of alveolar inflammation in each group was quantified according to criteria such as the area of tissue affected by inflammation. The scoring parameters included the degree of inflammatory cell infiltration in alveolar and interstitial spaces, disruption of lung tissue structural integrity, and the extent of collagen deposition.

### 4.4. Total RNA Extraction and RT-qPCR

The left lobe of the lung was collected from each mouse for total RNA extraction using RNAiso Plus, following the manufacturer’s instructions. The extracted RNA was quantified using a UV spectrophotometer. One microgram of total RNA was reverse transcribed into cDNA using a first-strand cDNA synthesis kit, following the provided protocol. Quantitative PCR (qPCR) was performed on a QuantStudio system using 2× SYBR Green qPCR Master Mix, and β-actin served as the internal control. The PCR reaction utilized a two-step protocol: 95 °C for 15 s, followed by 60 °C for 60 s, repeated for 40 cycles. Gene expression levels were calculated using the 2^−ΔΔCt^ method [31]. (Primers used in the study were synthesized by DynaTech Biotechnology Corporation and are listed in the Table 4).

### 4.5. Toxicity Study of ShSPI Single Administration in Mice

Specific pathogen-free (SIPF) grade ICR healthy mice (with an equal number of males and females, procured from Hunan Slaughter King Laboratory Animal Co., Changsha, China) were randomly divided into four experimental groups and received ShSPI via airway spray at doses of 0 mg/kg (control), 40 mg/kg, 80 mg/kg, or 160 mg/kg in a single administration. Following treatment, the mice were monitored for a 14-day observation period. On the fourteenth day, blood plasma samples were collected for precise measurement and analysis of hematological and biochemical parameters [32]. The specific process is as follows.

On day 14 post-dose, approximately 1.7–2.0 mL of whole blood was collected for hematological and biochemical analyses. Hematological parameters were assessed using an automated hematology analyzer, while biochemical parameters were evaluated with an automated biochemistry analyzer. Prior to blood collection, all mice were fasted for at least 12 h. For hematological analysis, 200 μL of whole blood was immediately transferred into EDTA-containing tubes. For biochemical analysis, 1.5 mL of whole blood was placed into serum separator tubes without anticoagulant and centrifuged to isolate serum.

At the end of the 14-day observation period, all mice underwent a comprehensive gross anatomical examination. Key organs, including the heart, liver, spleen, lungs, kidneys, brain, and gastrointestinal tract, were evaluated for changes in surface color, morphological structure, size, and tissue texture. Any significant pathological changes potentially induced by ShSPI treatment were identified and documented.

### 4.6. Pharmacokinetic Study of ShSPI in Rats

**Grouping and Sampling**: SIPF grade Sprague Dawley (SD) healthy rats (male, procured from Hunan Slaughter King Laboratory Animal Co.) were randomly divided into three groups, with three rats in each group. All groups received a single administration of the ShSPI and were fasted for 12 h prior to dosing. The groups included two inhalation groups (1 mg/kg and 2 mg/kg) and an intravenous tail vein injection group (1 mg/kg). Blood samples were collected from the subclavian vein at 5 min, 15 min, 30 min, 1 h, 2 h, 4 h, 8 h, and 24 h post-dosing. A 0.2 mL blood sample was transferred into pre-labeled EDTA-2K anticoagulant tubes and gently inverted to ensure thorough mixing of the anticoagulant with the blood. Samples were immediately placed on ice. Plasma was separated within 1 h by centrifugation at 6800 rpm for 5 min at 4 °C. The supernatant was collected and stored at −20 °C until analysis.

**Sample Preparation Before Analysis**: Sample preparation was conducted under yellow light in an ice-water bath. For each analysis, 30 μL of standard or experimental sample was mixed with 200 μL of working solution, while blank samples were prepared with an equivalent volume of methanol. The mixture was vortexed thoroughly and centrifuged at 5500 rpm for 10 min. A 150 μL aliquot of the supernatant was mixed with 150 μL of ultrapure water, and the final mixture was subjected to analysis.

**Chromatographic and Mass Spectrometry Conditions**: Chromatographic separation was performed using an AQ C18 column (2.1 × 50 mm, 5.0 μm). The injection volume was 10 μL. The mobile phase consisted of 0.1% formic acid in water (A) and methanol (B), with the following gradient program: 10% B at 0–0.01 min, 98% B at 0.01–1.8 min, 98% B at 1.8–2.5 min, and 10% B at 2.5–3.0 min. The flow rate was set at 0.6 mL/min. Mass spectrometry analysis was performed using an electrospray ionization (ESI) source in multiple reaction monitoring (MRM) mode [33,34].

### 4.7. Data Analysis

All data were analyzed using GraphPad Prism v10.3.0 (San Diego, CA, USA) and are shown as mean ± standard deviation (SD). For normal continuous variables, one-way analysis of variance (ANOVA) was used. Asterisks represent *p*-value classifications: * *p* < 0.05, ** *p* < 0.01, *** *p* < 0.001, and **** *p* < 0.0001. “ns” is “not significant”, which means there is no significant difference.

## Figures and Tables

**Table 1:** Indicators of hematological examination ($\bar{x}$ ± s, *n* = 6).

Name	40 mg/kg	80 mg/kg	160 mg/kg	Control
WBC (10^9^/L)	8.22 ± 3.30	6.66 ± 2.04	11.5 ± 4.13	6.48 ± 2.37
Neu (10^9^/L)	1.41 ± 0.69	1.35 ± 0.40	2.23 ± 1.28	1.30 ± 0.50
Lym (10^9^/L)	6.40 ± 2.49	4.66 ± 1.68	8.63 ± 2.79	4.80 ± 1.94
Mon (10^9^/L)	0.28 ± 0.13	0.38 ± 0.05	0.42 ± 0.21	0.25 ± 0.11
Eos (10^9^/L)	0.09 ± 0.05	0.22 ± 0.20	0.21 ± 0.19	0.10 ± 0.04
Bas (10^9^/L)	0.02 ± 0.01	0.03 ± 0.04	0.02 ± 0.01	0.02 ± 0.01
Neu% (%)	16.78 ± 1.90	21.23 ± 5.92	18.46 ± 5.30	20.73 ± 4.53
Lym% (%)	78.20 ± 2.18	68.93 ± 6.78	75.90 ± 6.54	73.00 ± 5.83
Mon% (%)	3.35 ± 0.74	6.16 ± 1.76 *	3.58 ± 0.74	4.03 ± 0.86
Eos% (%)	131 ± 0.77	3.10 ± 2.15	1.85 ± 1.23	1.80 ± 1.13
Bas% (%)	0.35 ± 0.28	0.56 ± 0.60	0.20 ± 0.12	0.43 ± 0.21
RBC (10^12^/L)	7.78 ± 0.23	7.49 ± 0.34	7.90 ± 0.31	7.52 ± 0.61
HGB (g/L)	123.50 ± 5.64	120.33 ± 6.83	126.66 ± 4.36	121.5 ± 10.55
HCT (%)	36.98 ± 1.32	35.93 ± 2.11	37.81 ± 1.30	36.23 ± 2.85
MCV (fL)	47.56 ± 0.95	47.95 ± 0.38	47.86 ± 65	48.16 ± 0.46
MCH (pg)	15.86 ± 0.43	16.08 ± 0.40	16.05 ± 0.40	16.16 ± 0.17
MCHC (g/L)	333.50±3.98	335.33 ± 6.28	335.33 ± 4.13	335.66 ± 4.63
RDW-CV (%)	12.70 ± 0.76	12.18 ± 0.22	12.83 ± 0.74	12.40 ± 40.41
RDW-SD (fL)	26.68 ± 1.26	25.75 ± 1.03	27.20 ± 1.81	26.21 ± 0.68
PLT (10^9^/L)	1326.50 ± 494.34	1272 ± 206.81	1511.2 ± 178.44	1319.8 ± 47.06
MPV (fL)	5.35 ± 0.18	5.48 ± 0.48	5.21 ± 0.21	5.31 ± 0.47
PDW	15.21 ± 0.17	15.33 ± 0.10	15.21 ± 0.07	15.18 ± 0.11
PCT (%)	0.71 ± 0.04	0.69 ± 0.09	0.78 ± 0.07	0.70 ± 10.07

Note: * indicates comparison with the blank control group, *p* < 0.05.

**Table 2:** Indicators of blood biochemistry ($\bar{x}$ ± s, *n* = 6).

Name	40 mg/kg	80 mg/kg	160 mg/kg	Control
ALT (U/L)	44.95 ± 22.02	38.58 ± 7.12	39.80 ± 7.79	49.76 ± 33.81
AST (U/L)	97.20 ± 13.33	92.95 ± 20.45	80.6 ± 16.79	83.03 ± 26.05
ALP (U/L)	85.25 ± 18.91	94.75 ± 22.33	98.6 ± 27.17	88.16 ± 23.88
r-GT (U/L)	0.38 ± 0.20	0.00 ± 1.01	0.31 ± 0.35	0.43 ± 0.49
ALBII (g/L)	29.13 ± 1.27	28.05 ± 1.53	30.1 ± 1.28	29.20 ± 2.56
TC (mmol/L)	2.09 ± 0.33	2.21 ± 0.61	2.26 ± 0.33	2.21 ± 0.45
CREA-S (μmol/L)	21.70 ± 4.99	23.10 ± 2.24	19.1 ± 3.98	18.7 ± 2.77
UREA (mmol/L)	17.07 ± 3.07	14.79 ± 3.07	13.6 ± 4.00	12.85 ± 1.75
CK (U/L)	236.4 ± 108.76	175.4 ± 69.9	156.4 ± 75.0	155.50 ± 104.86
Glu-G (mmol/L)	9.93 ± 1.48	9.71 ± 1.69	9.24 ± 1.33	9.90 ± 1.36
TG (mmol/L)	1.35 ± 0.50	1.21 ± 0.32	1.23 ± 0.35	1.33 ± 0.28
T-Bil-VII (μmol/L)	1.73 ± 0.80	1.47 ± 0.43	209 ± 0.75	1.68 ± 0.60
TPII (g/L)	46.81 ± 1.48	45.26 ± 1.62	48.2 ± 1.79	46.69 ± 2.61
AST/ALT	2.45 ± 0.79	2.45 ± 0.64	2.16 ± 0.81	2.21 ± 1.20
GLO2 (g/L)	17.67 ± 0.80	17.21 ± 0.82	18.0 ± 1.06	17.49 ± 1.03
A/G2 (g/L)	1.65 ± 0.11	1.63 ± 0.13	1.67 ± 0.12	1.67 ± 0.18
potassium ion (mmol/L)	4.82 ± 0.27	5.01 ± 0.46	4.68 ± 0.30	4.77 ± 0.33
sodium ion (mmol/L)	152.39 ± 1.75	150.7 ± 1.89	152.9 ± 1.60	152.75 ± 1.82
chloride ion (mmol/L)	106.80 ± 1.14	106.5 ± 1.26	106.5 ± 1.44	108.33 ± 0.68
calcium ionomer (mmol/L)	1.23 ± 0.01 *	1.24 ± 0.05 *	1.28 ± 0.03	1.33 ± 0.03
total calcium (mmol/L)	2.48 ± 0.02 *	2.49 ± 0.10 *	2.5 ± 0.06 *	2.67 ± 0.05
calcium binding (mmol/L)	1.23 ± 0.011	1.24 ± 0.05	1.28 ± 0.03	1.33 ± 0.03

Note: * denotes comparison with the blank control group, *p* < 0.05.

**Table 3:** Pharmacokinetic parameters of ShSPI in mice ($\bar{x}$ ± s, *n* = 3).

Pharmacokinetic Parameters	1 mg/kg ShSPI (Inhalation)	2 mg/kg ShSPI (Inhalation)
T_1/2_ (h)	1.98 ± 0.70	2.14 ± 0.663
C_max_ (ng·mL^−1^)	188.00 ± 64.40	347.00 ± 151.00
MRT_inf_ (h)	2.86 ± 0.725	3.1 ± 0.898
CI/F (ng·min^−1^·kg^−1^)	30.10 ± 15.20	29.00 ± 11.90
AUC_0–t_ (h·ng·mL^−1^)	580.00 ± 294.00	1169.00 ± 415.00
AUC_0–iaf_ (h·ng·mL^−1^)	641.00 ± 243.00	1265.00 ± 422.00
Tmax (h)	1.00	1.00

**Table 4:** Primer sequence.

Name	Upstream Primers (5′-3′)	Downstream Primers (5′-3′)
IL-10	GGTGAGAAGCTGAAGACCCT	ACACCTTGGTCTTGGAGCTT
MCP-1	GTCCCTGTCATGCTTCTGG	GCGTTAACTGCATCTGGCT
TGF-β	TTGCTTCAGCTCCACAGAGA	TGGTTGTAGAGGGCAAGGAC
IFN-γ	TGGCTGTTTCTGGCTGTTACTG	AATCAGCAGCGACTCCTTTTCC
IL-1β	GAAATGCCACCTTTTGACAGTG	TGGATGCTCTCATCAGGACAG
IL-6	CTGCAAGAGACTTCCATCCAG	AGTGGTATAGACAGGTCTGTTGG
β-actin	CACCATGTACCCAGGCATTG	CCTGCTTGCTGATCCACATC

## Data Availability

The original contributions presented in this study are included in the article. Further inquiries can be directed to the corresponding author(s).
